# P38 - Nut allergy and patterns of specific IgE components in two identical twins

**DOI:** 10.1186/2045-7022-4-S1-P93

**Published:** 2014-02-28

**Authors:** Chrys Tsakona

**Affiliations:** 1 Dudley Group of Hospitals NHS Foundation Truist, Dudley, United Kingdom

## 

Peanut allergy currently affects 1-2% of children in the developed countries. The role of genetics in its aetiology is unknown. For complex genetic traits, twin studies provide information on the relative contribution of genetic factors, as they decrease the relative confounding effects of the environmenl. The significantly higher concordance rate of peanut allergy among monozygotes suggests strongly that there is a significant genetic influence. On the other hand component-resolved diagnostics (CRD) utilize purified native or recombinant allergens to detect IgE sensitivity to individual allergen molecules and have gained growing importance. CRD allows to some extent discrimination between clinically significant and irrelevant specific IgE results and establishing of sensitisation patterns with particular prognostic outcomes.

Here is a case of identical male twins where the pattern of results, including that of distribution of IgE specific antibodies to individual molecules, is also identical. In the age of 4 yrs one had an ice cream with nut sprinkle and instantly developed lip swelling. They were both tested for nut and egg, as they had never had either before, were positive to both and avoided them ever since, although the other twin had once cooked egg accidentally and within few minutes developed abdominal pain and diarrhoea. They both have IgE antibodies to House Dust Mite, egg white and early and late tree pollens. Hazelnut is the predominant nut, followed by peanut and almond and all the rest negative. In Skin Prick Testing the strongest response was to peanut with moderate responses to almond and hazelnut and the rest negative. Analysis of the IgE antibody levels against individual nut components showed a predominance of Ara8, followed by Ara2 with Ara1 and Ara8 negative. The results arranged in a graph form two lines of identical shape. As far as we are concerned there is no similar report on identical twins and it would be useful to the geneticists as to what makes the same allergy not only manifested in both twins but also involving the same peanut molecules out of the 18 identified as being capable of binding allergen-specific IgE antibodies.

